# Clinical outcomes of COVID-19 in Wuhan, China: a large cohort study

**DOI:** 10.1186/s13613-020-00706-3

**Published:** 2020-07-31

**Authors:** Jiao Liu, Sheng Zhang, Zhixiong Wu, You Shang, Xuan Dong, Guang Li, Lidi Zhang, Yizhu Chen, Xiaofei Ye, Hangxiang Du, Yongan Liu, Tao Wang, SiSi Huang, Limin Chen, Zhenliang Wen, Jieming Qu, Dechang Chen

**Affiliations:** 1 Department of Critical Care Medicine, Ruijin Hospital, Shanghai Jiao Tong University School of Medicine, No.197 Ruijin 2nd Road, Shanghai, 200025 China; 2 Department of Surgical Intensive Care Unit, Huadong Hospital Affiliated to Fudan University, No.221 West Yan’an Road, Shanghai, 200040 China; 3 Department of Critical Care Medicine, Union Hospital, Tongji Medical College, Huazhong University of Science and Technology, No. 1277 Jiefang Avenue, Wuhan, 430022 China; 4 Tuberculosis and Respiratory Department, Wuhan Jinyin-tan Hospital, No.1 Yintan Road, Wuhan, 430023 China; 5 Department of Critical Care Medicine, Renmin Hospital of Wuhan University, No. 238 Jiefang Road, Wuhan, 430000 China; 6 Department of Health Statistics, Second Military Medical University, No.800 Xiangyin Road, Shanghai, 200433 China; 7 Department of Pulmonary and Critical Care Medicine, Ruijin Hospital, Shanghai Jiao Tong University School of Medicine, No.197 Ruijin 2nd Road, Shanghai, 200025 China

**Keywords:** Risk factors, COVID-19, Development, Severe, Mortality

## Abstract

**Background:**

Since December 2019, an outbreak of Coronavirus disease 2019 (COVID-19) caused by the severe acute respiratory syndrome coronavirus 2 (SARS-Cov-2) initially emerged in Wuhan, China, and has spread worldwide now. Clinical features of patients with COVID-19 have been described. However, risk factors leading to in-hospital deterioration and poor prognosis in COVID-19 patients with severe disease have not been well identified.

**Methods:**

In this retrospective, single-center cohort study, 1190 adult inpatients (≥ 18 years old) with laboratory-confirmed COVID-19 and determined outcomes (discharged or died) were included from Wuhan Infectious Disease Hospital from December 29, 2019 to February 28, 2020. The final follow-up date was March 2, 2020. Clinical data including characteristics, laboratory and imaging information as well as treatments were extracted from electronic medical records and compared. A multivariable logistic regression model was used to explore the potential predictors associated with in-hospital deterioration and death.

**Results:**

1190 patients with confirmed COVID-19 were included. Their median age was 57 years (interquartile range 47–67 years). Two hundred and sixty-one patients (22%) developed a severe illness after admission. Multivariable logistic regression demonstrated that higher SOFA score (OR 1.32, 95% CI 1.22–1.43, per score increase, *p *< 0.001 for deterioration and OR 1.30, 95% CI 1.11–1.53, per score increase, *p *= 0.001 for death), lymphocytopenia (OR 1.81, 95% CI 1.13–2.89 *p *= 0.013 for deterioration; OR 4.44, 95% CI 1.26–15.87, *p *= 0.021 for death) on admission were independent risk factors for in-hospital deterioration from not severe to severe disease and for death in severe patients. On admission D-dimer greater than 1 μg/L (OR 3.28, 95% CI 1.19–9.04, *p *= 0.021), leukocytopenia (OR 5.10, 95% CI 1.25–20.78), thrombocytopenia (OR 8.37, 95% CI 2.04–34.44) and history of diabetes (OR 11.16, 95% CI 1.87–66.57, *p *= 0.008) were also associated with higher risks of in-hospital death in severe COVID-19 patients. Shorter time interval from illness onset to non-invasive mechanical ventilation in the survivors with severe disease was observed compared with non-survivors (10.5 days, IQR 9.25–11.0 vs. 16.0 days, IQR 11.0–19.0 days, *p* = 0.030). Treatment with glucocorticoids increased the risk of progression from not severe to severe disease (OR 3.79, 95% CI 2.39–6.01, *p *< 0.001). Administration of antiviral drugs especially oseltamivir or ganciclovir is associated with a decreased risk of death in severe patients (OR 0.17, 95% CI 0.05–0.64, *p *< 0.001).

**Conclusions:**

High SOFA score and lymphocytopenia on admission could predict that not severe patients would develop severe disease in-hospital. On admission elevated D-dimer, leukocytopenia, thrombocytopenia and diabetes were independent risk factors of in-hospital death in severe patients with COVID-19. Administration of oseltamivir or ganciclovir might be beneficial for reducing mortality in severe patients.

## Introduction

Since December 2019, respiratory tract infection cases caused by virus occurred in Wuhan, Hubei Province, China [1, 2]. At first, a majority of cases was clustered around the local Huanan Seafood Wholesale Market, where wild animals were illegally sold. Then, the disease had rapidly spread from Wuhan to all over the China and to many foreign countries [3]. On Jan 7, the responsible novel coronavirus was identified by the Chinese Center for Disease Control and Prevention (CDC), and was subsequently named as severe acute respiratory syndrome coronavirus 2 (SARS-CoV-2; previously known as 2019-nCoV) by WHO, and pneumonia caused by 2019-nCoV was named COVID-19 [4]. The emerging virus was rapidly characterized as a novel member of the coronavirus family [5].

Some case series have demonstrated the clinical characteristics and epidemiological features of COVID-19 [6–8]. Clinical manifestations caused by SARS-CoV-2 varied, encompassing asymptomatic infection, pneumonia, acute respiratory distress syndrome (ARDS) and even death [6–8]. The mortality of patients with severe illness is extremely high [9]. However, risk factors leading to deterioration and poor outcome in severe COVID-19 patients have not been well described. In the present study, the clinical data of 1190 COVID-19 patients admitted in Wuhan Infectious Disease Hospital (discharge or death) were collected to analyze the clinical features and potential predictors for deterioration and/or death in COVID-19 patients. We paid close attention to the issues as below: first, comparing the clinical features between different severity and outcomes, shedding light on the risk factors for mortality and progression prediction; second, comparing the time interval to respiratory supports between survivors and non-survivors, exploring the preferable respiratory support to decrease mortality.

## Methods

### Study design and participants

This was a single-center, retrospective, observational study conducted from December 29, 2019, to February 28, 2020. A total of 1190 adult (18–94 years) patients with confirmed COVID-19 from Wuhan Infectious Disease Hospital were enrolled. All patients with confirmed COVID-19 enrolled in this study were diagnosed according to World Health Organization (WHO) interim guidance [10]. This study was approved by the Medicine Institutional Review Board of Wuhan Infectious Disease Hospital (KY-2020-03.01). Informed consents were waived from study participants.

### Data collection

The epidemiological, demographic, clinical, laboratory data were extracted mostly on admission from medical records. The collected information included age, sex, comorbidities, exposure history, oxygen support during hospitalization (nasal cannula, non-invasive mechanical ventilation, invasive mechanical ventilation or invasive medical ventilation with extracorporeal membrane oxygenation [ECMO]), symptoms onset on admission, vital signs, serum laboratory tests (including blood routine tests, blood chemical variables, procalcitonin, coagulation function tests), chest X-ray and computed tomographic (CT) scans, therapeutic strategy during hospitalization (antivirus treatment [ganciclovir, oseltamivir, arbidol, lopinavir and ritonavir, interferon], antibiotics [cefprozil, ceftriaxone, cefoperazone–sulbactam, piperacillin–tazobactam, biapenem, meropenem, vancomycin, linezolid, sulfamethoxazole, levofloxacin and moxifloxacin], glucocorticoids) and outcomes. Throat-swab specimens from patients with history of epidemiology and characteristics of virus pneumonia in chest CT or X-ray, were obtained. The time interval between two specimens was at least 24 h apart. Detection of 2019-nCoV nucleic acid was performed at the CDC before January 23, 2020, and subsequently at designated hospitals (Chinese Academy of Medical Sciences, Academy of Military Medical Sciences, and Wuhan Institute of Virology of the Chinese Academy of Sciences) as previously described. Patients with at least two consecutive times of positive results from high-throughput sequencing or real-time reverse-transcriptase polymerase chain reaction (RT-PCR) assay of nasal and pharyngeal swab specimens were confirmed with COVID-19. The included patients in the current study were all with determined laboratory results.

### Definition

COVID-19 diagnosis was according to WHO interim guidance [10]. The severity of COVID-19 was classified into mild, moderate, severe and critical type. The classification was assessed according to the diagnosis and treatment of COVID-19 guidelines (sixth version) published by the National Health Commission of China [11] (Additional file 1). Progressors were defined as mild or moderate patients who developed severe or critically illness during hospitalization. Non-progressors were defined as mild or moderate patients who never developed severe or critically illness during hospitalization. The disease onset was defined as the day when related symptoms first appeared.

### Endpoints

In the present study, the endpoints included in-hospital deterioration and/or death among those with severe disease. The time intervals from symptom onset or admission to high-flow nasal oxygen, non-invasive mechanical ventilation (NIV), invasive mechanical ventilation (IMV), extracorporeal membrane oxygenation (ECMO) were also recorded.

### Statistical analysis

Statistical analyses were performed using SPSS (version 24.0, SPSS Inc., Chicago, IL, USA) and SAS (version 9.3, SAS Institute, Cary, NC). Continuously normally distributed data were reported as mean (deviation) and compared using Student’s t test. Continuously non-normally distributed data were reported as median (interquartile range) and compared using Wilcoxon rank-sum test. Categorical data were presented as n (percentage) and compared using Chi-square test, Fisher’s exact test, and Cochran–Mantel–Haenszel test, as appropriate.

The potential risk factors for in-hospital deterioration (from not severe to severe disease) and death particularly in severe COVID-19 patients were determined using univariable and multivariable logistic regression model and displayed as odds ratio (OR) and 95% confidence interval (CI). Variables with a *p* value of 0.05 or less in the univariable analysis were considered as candidate variables in the multivariable analysis. Due to the statistical rule that the ratio of events and per variable should be ten or more, only 16 variables were finally selected based on the clinical importance. To exclude the possible bias introduced by missing data, we performed a sensitivity analysis using multiple imputations to account for missing data. Five imputations of complete data were generated and refitted into the multivariable logistic regression to test whether a selected variable remained to be the independent factor for illness deterioration and in-hospital mortality.

To evaluate the effects of anti-viral agents on clinical outcomes, we compared the difference of mortality and median survival time between patients who received or not received the anti-viral agents as follows: oseltamivir, ganciclovir, lopinavir–ritonavir, γ-interferon, arbidol. Kaplan–Meier curves and log-rank test were also used for survival analyses. To explore whether a specific anti-viral agent was independently associated with prolonged survival, we used multivariable Cox proportional-hazards model to compute the hazard ratio (HR) for each anti-viral agent by incorporating the same co-variables used in the multivariable logistic regression model for adjustment. A two-sided *p* value less than 0.05 was defined as statistically significant for all the analyses.

## Results

### Demographic and clinical characteristics

**Fig. 1:**
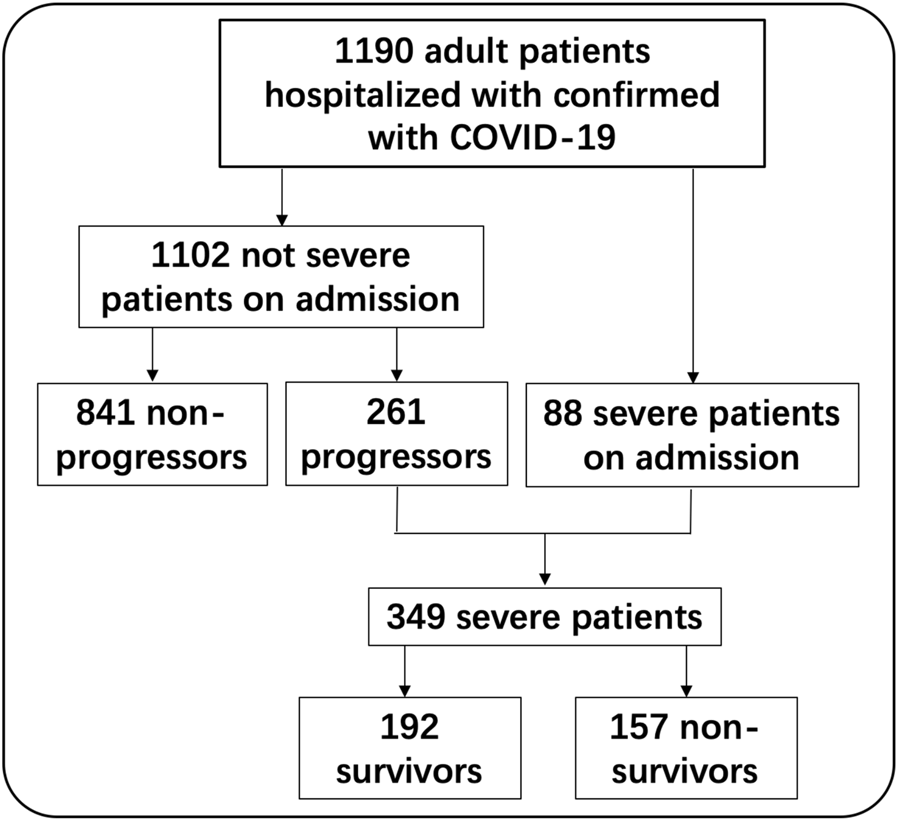
Flowchart in the present study

1190 patients with confirmed COVID-19 were recorded in Wuhan Infectious Disease Hospital during the study period, including 555 (46.6%) females and 635 males (53.4%), with an average age of 57 years (47–67). The flowchart of the current study is shown in Fig. 1. Demographic and clinical details were obtained for all the patients (Table 1). In total, 131 (11.4%) patients had a history of exposure to the Huanan seafood market, 132 (11.2%) were household clustered, and 16 (1.4%) were medical staff. The most commonly self-reported symptoms on admission were fever (*n* = 971, 81.9%), cough (*n* = 879, 74.2%), dyspnea (*n* = 548, 46.3%), fatigue (*n* = 434, 36.7%) and sputum production (*n* = 417, 35.2%). 441 (37.1%). Patients had comorbidities, including chronic obstructive pulmonary disease (*n* = 22, 1.9%), diabetes (*n* = 144, 12.2%), hypertension (*n* = 308, 26.1%), chronic cardiac disease (*n* = 86, 7.3%), chronic kidney disease (*n* = 30, 2.6%), chronic liver disease (*n* = 40, 3.4%), stroke (*n* = 39, 3.3%), malignancy (*n* = 34, 2.9%), immunosuppression (*n* = 24, 2.0%), and tuberculosis (*n* = 15, 1.3%).

**Table 1:** Clinical characteristics, radiographic, laboratory results of patients with COVID-19

	All patients (*n* = 1190)	Survivor (*n* = 1033)	Non-survivor (*n* = 157)	*p* value
Age				
Median (IQR), year	57 (47, 67)	56 (46, 65)	69 (62, 77)	< 0.0001
Sex, *n* (%)				
Female	555 (46.6)	498 (48.2)	57 (36.3)	0.0053
Male	635 (53.4)	535 (51.8)	100 (63.7)	
Smoking, *n* (%)	45 (4.5)	40 (4.6)	5 (3.8)	1
Drinking, *n* (%)	48 (4.6)	43 (4.7)	5 (3.9)	0.6901
Epidemic disease history, *n* (%)				
Influenza A				
Negative	1131 (96.5)	987 (96.7)	144 (94.8)	0.4308
Positive	19 (1.6)	15 (1.5)	4 (2.6)	
Unchecked or unknown	22 (1.9)	18 (1.8)	4 (2.6)	
Influenza B				
Negative	1133 (96.6)	990 (97.0)	143 (94.1)	0.1257
Positive	18 (1.5)	13 (1.2)	5 (3.3)	
Unchecked or unknown	22 (1.9)	18 (1.8)	4 (2.6)	
Exposure history, *n* (%)				
Huanan seafood market	131 (11.4)	125 (12.5)	6 (4.1)	0.0028
Wuhan exposure	1119 (94.7)	968 (94.2)	151 (98.1)	0.0451
Other parts of Hubei	56 (5.0)	54 (5.5)	2 (1.4)	0.0373
Contact with wildlife	17 (1.5)	17 (1.8)	0 (0.0)	0.2238
Medical staff	16 (1.4)	16 (1.6)	0 (0.0)	0.2446
Clustered cases	132 (11.2)	118 (11.5)	14 (9.2)	0.6726
Any comorbidity, *n* (%)	441 (37.1%)	345 (33.4%)	96 (61.15%)	< 0.0001
Chronic obstructive pulmonary disease	22 (1.9)	14 (1.4)	8 (5.3)	1
Diabetes	144 (12.2)	105 (10.2)	39 (25.5)	< 0.0001
Hypertension	308 (26.1)	244 (23.8)	64 (41.8)	< 0.0001
Chronic cardiac disease	86 (7.3)	61 (6.0)	25 (16.3)	< 0.0001
Chronic kidney disease	30 (2.6)	24 (2.4)	6 (3.9)	0.38
Chronic liver disease	40 (3.4)	32 (3.1)	8 (5.2)	0.1779
Stroke	39 (3.3)	28 (2.7)	11 (7.2)	0.0041
Malignancy	34 (2.9)	26 (2.5)	8 (5.2)	0.1115
Immunosuppression	24 (2.0)	15 (1.5)	9 (5.9)	0.0009
Tuberculosis	15 (1.3)	10 (1.4)	5 (3.3)	0.0475
Signs and symptoms at admission, *n* (%)				
Fever	971 (81.9)	834 (80.9)	137 (89.0)	0.0152
Median highest temperature (IQR), °C	38.5 (38.0, 39.0)	38.5 (38.0, 39.0)	38.5 (38.0, 39.0)	0.0233
Nasal congestion	11 (0.9)	8 (0.8)	3 (2.0)	1
Nasal discharges	16 (1.4)	13 (1.3)	3 (2.0)	0.7521
Sneeze	5 (0.4)	4 (0.4)	1 (0.7)	0.5019
Sore throat	39 (3.3)	36 (3.5)	3 (2.0)	0.3171
Cough	879 (74.2)	751 (72.8)	128 (83.7)	0.0041
Sputum production	417 (35.2)	352 (34.1)	65 (42.5)	0.0438
Dyspnoea	548 (46.3)	439 (42.6)	109 (71.2)	< 0.0001
Chest pain	62 (5.3)	56 (5.5)	6 (3.9)	0.427
Hemoptysis	14 (1.2)	11 (1.1)	3 (2.0)	0.5846
Headache	61 (5.2)	59 (5.8)	2 (1.3)	0.0204
Myalgia	133 (11.3)	116 (11.3)	17 (11.1)	0.937
Fatigue	434 (36.7)	369 (35.9)	65 (42.5)	0.1128
Gastrointestinal symptoms	214 (18.2)	189 (18.4)	25 (16.3)	0.5333
Eye symptoms	23 (2.0)	22 (2.2)	1 (0.7)	0.3502
Ronchi	57 (4.8)	47 (4.6)	10 (6.5)	0.2953
Crackles	170 (14.4)	143 (13.9)	27 (17.5)	0.2265
Systolic pressure				
Median (IQR), mmHg	122 (111, 135)	122 (110, 134)	130.5 (117, 144)	0.0002
Diastolic pressure				
Median (IQR), mmHg	80 (72, 87)	80 (73, 87)	80 (72, 87)	0.0944
Heart rate				
Median (IQR), bpm	86 (79, 96)	86 (78, 96)	89 (82, 102)	< 0.0001
Respiratory rate				
Median (IQR), bpm	22 (20, 25)	21 (20, 25)	23 (20, 28)	0.9936
SOFA	3 (1, 5)	2 (1, 4)	10 (6, 18)	< 0.0001
APACHEII	3 (1, 6)	3 (1, 5)	10.5 (8, 17)	< 0.0001
Laboratory findings				
Leucocytes (IQR-10^9^/L)	6.3 (4.6, 9.1)	6.0 (4.5, 8.1)	15.5 (8.9, 21.9)	< 0.0001
Distribution, *n* (%)				
< 4	185 (16.1)	171 (16.9)	14 (10.1)	< 0.0001
4–10	726 (63.0)	702 (69.2)	24 (17.3)	
> 10	242 (21.0)	141 (13.9)	101 (72.6)	
Neutrophils (IQR-10^9^/L)	4.4 (2.9, 7.3)	4.1 (2.8, 6.2)	14.7 (9.9, 20.3)	< 0.0001
Distribution, *n* (%)				
< 1.8	65 (5.8)	61 (6.1)	4 (3.1)	< 0.0001
1.8–6.3	715 (63.2)	702 (70.2)	13 (10.0)	
> 6.3	351 (31.0)	237 (23.7)	114 (87.0)	
Lymphocytes (IQR-10^9^/L)	1.2 (0.7, 1.6)	1.2 (0.9, 1.6)	0.5 (0.3, 0.9)	< 0.0001
Distribution, *n* (%)				
< 0.8	315 (28.0)	221 (22.2)	94 (72.9)	< 0.0001
≥ 0.8	809 (72.0)	774 (77.8)	35 (27.1)	
CD3 (IQR-/μL)	618 (427, 964)	647 (468, 991)	367 (267, 409)	< 0.0001
CD4 (IQR-/μL)	366 (242, 594)	388 (275, 645)	211 (275, 645)	< 0.0001
CD8 (IQR-/μL)	235 (138, 337)	242 (156, 356)	129 (87, 144)	< 0.0001
Hemoglobin (IQR-g/L)	120 (109.0, 130.0)	120 (110.0, 130.0)	120 (103.0, 133.0)	0.4723
Distribution, *n* (%)				
≤ 90	54 (4.7)	7 (3.7)	17 (12.8)	< 0.0001
> 90	1092 (95.3)	976 (96.3)	116 (87.3)	
Platelets (IQR-10^9^/L)	193 (143.0, 250.0)	201 (154.0, 256.0)	90.5 (50.0, 165.0)	< 0.0001
Distribution, *n* (%)				
< 100	122 (10.6)	49 (4.8)	73 (52.9)	< 0.0001
≥ 100	1029 (89.4)	964 (95.2)	65 (47.1)	
Prothrombin time (IQR-s)	11.5 (10.7, 12.6)	11.4 (10.6, 12.3)	14 (12.4, 17.5)	< 0.0001
Distribution, *n* (%)				
< 10.5	201 (18.0)	197 (20.0)	4 (3.0)	< 0.0001
10.5–13.5	763 (68.2)	711 (72.3)	52 (38.8)	
> 13.5	154 (13.8)	76 (7.7)	78 (58.2)	
Activated-partial thromboplastin time (IQR-s)	27.7 (24.3, 32.5)	27.2 (24.2, 31.8)	33.4 (26.1, 38.9)	< 0.0001
Distribution, *n* (%)				
< 21	68 (6.1)	61 (6.2)	7 (5.4)	< 0.0001
21–37	927 (83.4)	847 (86.3)	80 (62.0)	
> 37	116 (10.4)	74 (7.5)	42 (32.6)	
Thrombin time (IQR, s)	17.9 (16.7, 20.6)	17.8 (16.7, 20.4)	18.4 (17.1, 23.0)	0.0054
Distribution, *n* (%)				
< 13	8 (0.7)	8 (0.8)	0 (0.0)	0.0321
13–21	842 (75.9)	753 (76.7)	89 (69.5)	
> 21	260 (23.4)	221 (22.5)	39 (30.5)	
D-dimer (IQR, μg/mL)	0.9 (0.4, 2.5)	0.8 (0.4, 1.6)	17.8 (4.5, 56.5)	< 0.0001
Distribution, *n* (%)				
≤ 0.5	323 (29.6)	319 (33.2)	4 (3.1)	< 0.0001
0.5–1	279 (25.6)	270 (28.1)	9 (6.9)	
> 1	489 (44.8)	371 (38.7)	118 (90.9)	
Total bilirubin (IQR, μmol/L)	13 (10.1, 17.7)	12.4 (9.8, 16.1)	24.9 (16.6, 36.1)	< 0.0001
Distribution, *n* (%)				
≤ 26	1005 (90.0)	932 (94.8)	73 (54.5)	< 0.0001
> 26	112 (10.0)	51 (5.2)	61 (45.5)	
Alanine aminotransferase (IQR-U/L)	42 (25.0, 66.0)	40 (24.0, 62.0)	47 (31.0, 84.0)	0.0003
Distribution, *n* (%)				
≤ 40	559 (48.8)	508 (50.2)	51 (37.8)	0.0065
> 40	587 (51.2)	503 (49.8)	84 (62.2)	
Aspartate aminotransferase (IQR-U/L)	35 (26.0, 51.0)	33 (25.0, 46.0)	58 (44.0, 109.0)	< 0.0001
Distribution, *n* (%)				
≤ 40	702 (61.2)	680 (67.3)	22 (16.1)	< 0.0001
> 40	445 (38.8)	330 (32.7)	115 (83.9)	
Albumin (IQR, g/L)	31.3 (28.0, 34.7)	32 (29.0, 35.2)	26.15 (24.3, 28.3)	< 0.0001
Distribution, *n* (%)				
< 40	1106 (96.2)	966 (95.6)	140 (100.0)	0.0144
40–55	41 (3.6)	41 (4.1)	0 (0.0)	
> 55	3 (0.3)	3 (0.3)	0 (0.0)	
Serum prealbumin (IQR-g/L)	125 (80.0, 187.0)	137 (91.0, 194.0)	48.5 (29.5, 75.0)	< 0.0001
Distribution, *n* (%)				
< 200	874 (79.2)	748 (76.7)	126 (98.4)	< 0.0001
200–430	229 (20.8)	227 (23.3)	2 (1.6)	
Blood urea nitrogen (IQR-mmol/L)	5.2 (4.1, 6.8)	4.97 (4.0, 6.2)	13.2 (7.7, 20.3)	< 0.0001
Distribution, *n* (%)				
< 3.1	81 (7.1)	81 (8.0)	0 (0.0)	< 0.0001
3.1–8	865 (75.7)	827 (82.1)	38 (28.4)	
> 8	196 (17.2)	100 (9.9)	96 (71.6)	
Serum creatinine (IQR, μmol/L)	72.6 (59.6, 88.6)	71.5 (59.0, 84.3)	107.8 (69.2, 196.7)	<0.0001
Distribution, *n* (%)				
> 133	84 (7.4)	32 (3.2)	52 (39.4)	< 0.0001
≤ 133	1051 (92.6)	971 (96.8)	80 (60.6)	
Creatine kinase (IQR-U/L)	78 (51.0, 151.0)	73 (49.0, 132.5)	240 (101.0, 553.0)	< 0.0001
Distribution, *n* (%)				
< 50	243 (23.7)	236 (25.7)	7 (6.6)	< 0.0001
50–310	676 (65.9)	619 (67.3)	57 (53.8)	
> 310	107 (10.4)	65 (7.0)	42 (39.6)	
Creatine kinase isoenzyme MB (IQR-U/L)	14 (10.0, 18.0)	13 (10.0, 17.0)	24 (18.0, 47.0)	< 0.0001
Distribution, *n* (%)				
≤ 24	960 (88.4)	896 (93.3)	64 (50.8)	< 0.0001
> 24	126 (11.6)	64 (6.7)	62 (49.2)	
C-reactive protein (IQR, mg/L)	30.1 (5.7, 92.0)	22.5 (4.3, 67.2)	160 (124.2, 177.1)	< 0.0001
Distribution, *n* (%)				
≤ 6.9	290 (28.4)	287 (32.1)	3 (2.3)	< 0.0001
> 6.9	731 (71.6)	606 (67.9)	125 (97.7)	
Serum amyloid protein A (IQR-mg/L)	190.8 (34.3, 275.9)	178.6 (25.6, 270.3)	260.1 (188.9, 284.0)	< 0.0001
Distribution, *n* (%)				
≤ 10	151 (15.8)	149 (17.5)	2 (1.9)	< 0.0001
> 10	805 (84.2)	702 (82.5)	103 (98.1)	
Serum ferritin (IQR-ng/mL)	406.1 (137.2, 800.8)	384.8 (146.0, 711.8)	616.6 (38.7, 2000.0)	0.0099
Distribution, *n* (%)				
< 21.8	36 (4.7)	32 (4.9)	4 (3.6)	0.7535
21.8–274.6	263 (34.2)	224 (34.1)	39 (34.8)	
> 274.6	470 (61.1)	401 (61.0)	69 (61.6)	
Interleukin-6 (IQR-pg/mL)	14.45 (8.0, 416.0)	13.2 (7.7, 366.2)	31.9 (11.1, 1487.0)	< 0.0001
Distribution, *n* (%)				
≤ 7	28 (3.4)	25 (3.5)	3 (2.8)	0.909
> 7	789 (96.6)	684 (96.5)	105 (97.2)	
Radiologic findings				
Abnormalities, *n* (%)				
Ground-glass opacity	1027 (92.3)	910 (92.3)	117 (92.1)	0.9474
Pulmonary consolidation	194 (17.4)	155 (15.7)	39 (30.7)	< 0.0001
Pulmonary interstitial abnormalities	700 (63.0)	609 (61.8)	91 (71.7)	0.0309
Pneumothorax	31 (2.8)	24 (2.4)	7 (5.5)	0.0901
Pleural effusion	49 (4.4)	43 (4.4)	6 (4.7)	0.851

*SOFA* Sequential Organ Failure Assessment, *APACHEII* Acute Physiology and Chronic Health Evaluation II, *ICU* intensive care unit, *MV* mechanical ventilation

On admission, the conditions of most patients (1102, 92.6%) were not severe, of whom 261 (22.7%) patients progressed into severe disease after admission (median 12 days, IQR 2–15 days). Compared with non-progressors, patients that progressed into a severe disease were older (62 vs. 55 year, *p *< 0.0001) and more male (60.1% vs. 51.0%, *p *= 0.0097), had more comorbidities such as diabetes (16.5% vs. 9.8%, *p *= 0.0033), hypertension (29.7% vs. 22.6%, *p *= 0.0208), stroke (7.3% vs. 2.2%, *p *= 0.0001), malignancy (4.7% vs. 2.0%, *p *= 0.0214) and immunosuppression (4.7% vs. 0.8%, *p *= 0.0001), and showed more severe initial symptoms, such as dyspnea (60.1% vs. 39.7%, *p *< 0.0001) and higher heart rate (89 vs. 85 bpm, *p *= 0.0002) (Table 2).

**Table 2:** Treatments and clinical outcomes of patients with COVID-19

	All patients (*n* = 1190)	Survivor (*n* = 1033)	Non-survivor (*n* = 157)	*p* value
Treatments, *n* (%)				
Antibiotic	977 (87.7)	859 (87.0)	118 (92.9)	0.0575
Antifungal	50 (4.5)	35 (3.6)	15 (11.8)	< 0.0001
Antiviral	681 (61.1)	626 (63.4)	55 (43.3)	< 0.0001
Glucocorticoids	289(25.9)	213 (21.6)	76 (59.8)	< 0.0001
Oxygen therapy, *n* (%)				< 0.0001
None	203 (17.1)	203 (19.7)	0 (0.0)	
Nasal cannula	792(66.6)	776 (75.1)	16(10.2)	
Mask oxygen	27 (2.3)	19 (1.9)	7 (4.5)	
High-flow nasal cannula	60 (5.0)	24 (2.3)	36 (22.9)	
Non-invasive mechanical ventilation	62 (5.2)	4 (0.4)	58 (36.9)	
Invasive mechanical ventilation	42 (3.5)	6 (0.6)	36 (22.9)	
ECMO	4(0.3)	0	4 (2.6)	
Outcomes				
Duration of MV (IQR), days	5 (2.0, 8.0)	6 (5.0, 9.0)	4 (2.0,8.0)	0.1563
Duration of ICU stay (IQR), days	6 (3.0, 10.5)	7 (4.0, 11.0)	5 (2.0, 9.0)	0.0522
Duration of in-hospital stay (IQR), days	11 (7.0, 14.5)	11 (8.0, 15.0)	8 (4.0, 12.0)	< 0.0001
In-hospital mortality, *n* (%)	157 (13.2)	0 (0.0)	157 (100.0)	< 0.0001

*ECMO* extracorporeal membrane oxygenation, *ICU* intensive care unit, *MV* mechanical ventilation

A total of 349 severe patients were found including 88 patients who were severe on admission and 261 patients who had an initial not severe disease that progressed to a severe disease during their hospital stay. There were 157 (45.0%) deaths among the 349 severe patients. Non-survivors were older than in survivors (69 vs. 57 year, *p *< 0.0001). There were more comorbidities including diabetes (25.5% vs. 12.2%, *p *= 0.0015), hypertension (41.8% vs. 29.0%, *p *= 0.0127) and chronic cardiac disease (16.3% vs. 6.3%, *p *= 0.0029) in the non-survivor group than in the survivor group. The major in-hospital complication rates were higher in the non-survivor group than in the survivor group (Additional file 2: Table S1). Compared with survivors, non-survivors presented with more dyspnea (71.2% vs. 55.2%, *p *= 0.0023) on admission (Table 3).

**Table 3:** Clinical characteristics, radiographic, laboratory results of the study patients

	Not severe patients at admission **(***n* = 1102)	Non-progressors (*n* = 841)	Progressors (*n* = 261)	*p* value	Severe patients (*n* = 349)	Survivor (*n* = 192)	Non-survivor (*n* = 157)	*p* value
Age								
Median (IQR), year	56 (46, 66)	55 (45, 65)	62 (52, 70)	< 0.0001	63 (53, 72)	57 (48, 66)	69 (62, 77)	< 0.0001
Sex, *n* (%)								
Female	516 (46.8)	412 (49.0)	104 (39.9)	0.0097	143 (41.0)	86 (44.8)	57 (36.3)	0.1088
Male	586 (53.2)	429 (51.0)	157 (60.1)		206 (59.0)	106 (55.2)	100 (63.7)	
Smoking, *n* (%)	40 (4.3)	25 (3.5)	15 (7.2)	1	20 (7.0)	15 (9.9)	5 (3.8)	1
Drinking, *n* (%)	44 (4.6)	25 (3.3)	19 (8.8)	0.0006	23 (7.9)	18 (11.0)	5 (3.9)	0.0251
Epidemic disease history, *n* (%)								
Influenza A								
Negative	1045 (96.3)	799 (96.3)	246 (96.5)	0.4315	332 (97.1)	188 (98.9)	144 (94.8)	0.0421
Positive	19 (1.8)	13 (1.6)	6 (2.3)		6 (1.7)	2 (1.1)	4 (2.6)	
Unchecked or unknown	21 (1.9)	18 (2.1)	3 (1.2)		4 (1.2)	0 (0.00)	4 (2.6)	
Influenza B								
Negative	1048 (96.5)	800 (96.3)	248 (97.3)	0.6044	333 (97.4)	190 (100.0)	143 (94.1)	0.0032
Positive	17 (1.6)	13 (1.5)	4 (1.6)		5 (1.5)	0 (0.0)	5 (3.3)	
Unchecked or unknown	21 (1.9)	18 (2.2)	3 (1.1)		4 (1.1)	0 (0.0)	4 (2.6)	
Exposure history, *n* (%)								
Huanan seafood market	126 (11.9)	96 (11.8)	30 (12.2)	0.8499	35 (10.5)	29 (15.7)	6 (4.1)	0.0006
Wuhan exposure	1032 (94.3)	788 (94.0)	244 (95.3)	0.4385	331 (96.2)	180 (94.7)	151 (98.1)	0.1089
Other parts of Hubei	55 (5.2)	44 (5.4)	11 (4.6)	0.6121	12 (3.8)	10 (5.7)	2 (1.4)	0.0458
Contact with wildlife	17 (1.6)	10 (1.2)	7 (3.0)	0.1169	7 (2.2)	7 (4.0)	0 (0.0)	0.0404
Medical staff	16 (1.5)	16 (1.9)	0 (0.0)	0.0608	0 (0.0)	0 (0.0)	0 (0.0)	1
Clustered cases	125 (11.5)	88 (10.6)	37 (14.5)	0.1597	44 (12.8)	30 (15.8)	14 (9.2)	0.1736
Any comorbidity, *n* (%)	383 (34.8)	265 (31.5)	118 (45.2)	< 0.0001	176 (50.4)	80 (41.7)	96 (61.2)	0.0003
Chronic obstructive pulmonary disease	18 (1.7)	9 (1.1)	9 (3.6)	0.2117	13 (3.8)	5 (2.7)	8 (5.3)	1
Diabetes	124 (11.4)	82 (9.8)	42 (16.5)	0.0033	62 (18.1)	23 (12.2)	39 (25.5)	0.0015
Hypertension	265 (24.3)	189 (22.6)	76 (29.7)	0.0208	119 (34.7)	55 (29.0)	64 (41.8)	0.0127
Chronic cardiac disease	70 (6.4)	49 (5.9)	21 (8.2)	0.1823	37 (10.8)	12 (6.3)	25 (16.3)	0.0029
Chronic kidney disease	29 (2.7)	20 (2.4)	9 (3.5)	0.3326	10 (2.9)	4 (2.1)	6 (3.9)	0.5022
Chronic liver disease	37 (3.4)	28 (3.4)	9 (3.5)	0.9165	12 (3.5)	4 (2.1)	8 (5.2)	0.1132
Stroke	36 (3.3)	18 (2.2)	18 (7.3)	0.0001	21 (6.1)	10 (5.3)	11 (7.2)	0.4595
Malignancy	29 (2.7)	17 (2.0)	12 (4.7)	0.0214	17 (5.0)	9 (4.7)	8 (5.2)	0.8347
Immunosuppression	19 (1.8)	7 (0.8)	12 (4.7)	0.0001	17 (5.0)	8 (4.2)	9 (5.9)	0.4695
Tuberculosis	14 (1.3)	8 (1.0)	6 (2.4)	0.1589	7 (2.1)	2 (1.1)	5 (3.3)	0.2858
Signs and symptoms at admission, *n* (%)								
Fever	889 (81.0)	671 (80.0)	218 (84.2)	0.1329	300 (86.7)	163 (84.9)	137 (89.0)	0.2684
Median highest temperature (IQR) °C	38.5 (38.0, 39.0)	38.4 (38.0, 39.0)	38.55 (38.0, 39.0)	0.4549	38.5 (38.0, 39.0)	38.5 (38.0, 39.0)	38.5 (38.0, 39.0)	0.0554
Nasal congestion	11 (1.0)	5 (0.6)	6 (2.3)	1	6 (1.7)	3 (1.6)	3 (2.0)	1
Nasal discharges	16 (1.5)	10 (1.2)	6 (2.3)	0.309	6 (1.7)	3 (1.6)	3 (2.0)	1
Sneeze	5 (0.5)	2 (0.2)	3 (1.2)	0.1646	3 (0.9)	2 (1.0)	1 (0.7)	1
Sore throat	36 (3.3)	31 (3.7)	5 (1.9)	0.1611	8 (2.3)	5 (2.6)	3 (2.0)	0.9725
Cough	810 (73.8)	600 (71.4)	210 (81.4)	0.0015	279 (80.9)	151 (78.7)	128 (83.7)	0.2394
Sputum production	391 (35.6)	282 (33.6)	109 (42.3)	0.0113	135 (39.1)	70 (36.5)	65 (42.5)	0.2546
Dyspnoea	488 (44.5)	333 (39.7)	155 (60.1)	< 0.0001	215 (62.3)	106 (55.2)	109 (71.2)	0.0023
Chest pain	57 (5.2)	46 (5.5)	11 (4.3)	0.4294	16 (4.6)	10 (5.2)	6 (3.9)	0.5723
Hemoptysis	11 (1.0)	8 (1.0)	3 (1.2)	1	6 (1.7)	3 (1.6)	3 (2.0)	1
Headache	60 (5.5)	52 (6.3)	8 (3.1)	0.0527	9 (2.6)	7 (3.7)	2 (1.3)	0.3106
Myalgia	126 (11.6)	88 (10.6)	38 (14.7)	0.0684	45 (13.0)	28 (14.6)	17 (11.1)	0.3414
Fatigue	397 (36.3)	298 (35.6)	99 (38.5)	0.3878	136 (39.5)	71 (37.2)	65 (42.5)	0.3167
Gastrointestinal symptoms	200 (18.3)	163 (19.5)	37 (14.3)	0.059	51 (14.8)	26 (13.5)	25 (16.3)	0.4669
Eye symptoms	23 (2.1)	16 (1.9)	7 (2.7)	0.442	7 (2.0)	6 (3.1)	1 (0.7)	0.2175
Rhonchi	51 (4.6)	33 (3.9)	18 (7.0)	0.0438	24 (6.9)	14 (7.3)	10 (6.5)	0.7715
Crackles	150 (13.7)	111 (13.2)	39 (15.1)	0.454	59 (17.1)	32 (16.7)	27 (17.5)	0.8315
Systolic pressure								
Median (IQR), mmHg	122 (110, 134)	121 (110, 133)	123 (112, 136)	0.2233	126 (115, 139)	123 (112, 136)	130.5 (117, 144)	0.0201
Diastolic pressure								
Median (IQR), mmHg	80 (72, 87)	80 (72, 88)	80 (72, 87)	0.173	80 (73, 87)	80 (75, 87)	80 (72, 87)	0.2204
Heart rate								
Median (IQR), bpm	86 (79, 96)	85 (78, 95)	89 (80, 100)	0.0002	89 (80, 100)	88 (80, 98)	89 (82, 102)	0.1859
Respiratory rate								
Median (IQR), bpm	21 (20, 25)	21 (20, 24)	22 (20, 26)	0.3707	23 (20, 28)	22 (20, 28)	23 (20, 28)	0.2702
SOFA	2 (1, 5)	2 (0, 14)	4 (2, 8)	< 0.0001	5 (3, 10)	3 (2, 5)	10 (6, 18)	< 0.0001
APACHEII	3 (1, 5)	3 (1, 5)	5 (3, 8)	< 0.0001	6 (3, 10)	5 (2, 7)	10.5 (8, 17)	< 0.0001
Laboratory findings								
Leucocytes- (IQR-10^9^/L)	6.1 (4.5, 8.5)	5.8 (4.5, 7.8)	8.1 (5.0, 13.6)	< 0.0001	9.4 (5.8, 15.6)	7.3 (4.8, 10.2)	15.5 (8.9, 21.9)	< 0.0001
Distribution, *n* (%)								
<4	180 (16.8)	145 (17.5)	35 (14.4)	< 0.0001	40 (12.3)	26 (14.0)	14 (10.0)	< 0.0001
4–10	707 (66.0)	593 (71.6)	114 (46.7)		133 (40.9)	109 (58.6)	24 (17.3)	
>10	185 (17.2)	90 (10.9)	95 (38.9)		152 (46.8)	51 (27.4)	101 (72.7)	
Hemoglobin (IQR-g/L)	121 (110.0, 131.0)	121 (110.0, 130.0)	119 (108.0, 131.0)	0.1872	118 (107.0, 130.0)	116.5 (108.0, 129.0)	120 (103.0, 133.0)	0.5845
Distribution, *n* (%)								
≤ 90	47 (4.4)	27 (3.3)	20 (8.3)	0.0008	27 (8.5)	10 (5.4)	17 (12.8)	0.0191
>90	1021 (95.6)	800 (96.7)	221 (91.7)		292 (91.5)	176 (94.6)	116 (87.2)	
Platelets (IQR-10^9^/L)	196 (147.0, 253.0)	204 (159.0, 260.0)	153 (105.0, 216.0)	< 0.0001	151.5 (90.5, 208.0)	179.5 (140.0, 241.0)	90.5 (50.0, 165.0)	< 0.0001
Distribution, *n* (%)								
<100	91 (8.5)	34 (4.1)	57 (23.5)	< 0.0001	88 (27.2)	15 (8.1)	73 (52.9)	< 0.0001
≥ 100	979 (91.5)	793 (95.9)	186 (76.5)		236 (72.8)	171 (91.9)	65 (47.1)	
Neutrophils (IQR-10^9^/L)	4.2 (2.8, 6.6)	3.8 (2.7, 5.7)	7.0 (3.6, 13.3)	< 0.0001	8.3 (4.6, 15.1)	5.6 (3.3, 9.1)	14.7 (9.9, 20.3)	< 0.0001
Distribution, *n* (%)								
< 1.8	65 (6.2)	57 (6.9)	8 (3.4)	< 0.0001	8 (2.6)	4 (2.3)	4 (3.1)	< 0.0001
1.8–6.3	703 (66.5)	606 (73.5)	97 (41.6)		109 (35.5)	96 (54.5)	13 (9.9)	
> 6.3	289 (27.3)	161 (19.6)	128 (55.0)		190 (61.9)	76 (43.2)	114 (87.0)	
Lymphocytes (IQR-10^9^/L)	1.2 (0.8, 1.6)	1.3 (0.9, 1.7)	0.8 (0.5, 1.3)	< 0.0001	0.8 (0.4, 1.2)	1.0 (0.6, 1.4)	0.5 (0.3, 0.9)	< 0.0001
Distribution, *n* (%)								
< 0.8	269 (25.6)	154 (18.8)	115 (49.6)	< 0.0001	161 (53.0)	67 (38.3)	94 (72.9)	< 0.0001
≥ 0.8	783 (74.4)	666 (81.2)	117 (50.4)		143 (47.0)	108 (61.7)	35 (27.1)	
CD3 (IQR-/μL)	626 (445, 964)	710 (470, 1132)	522 (367, 636.)	< 0.0001	522 (364, 659)	562 (427, 793)	367 (267, 409)	0.0004
CD4 (IQR-/μL)	368 (252, 612)	416 (283, 730)	292 (207, 432)	0.0006	289 (185, 432)	353 (261, 489)	211 (145, 248)	0.0003
CD8 (IQR-/μL)	237 (139, 337)	269 (188, 400)	155 (114, 252)	< 0.0001	155 (116, 252)	207 (128, 288)	129 (87, 144)	0.0044
Prothrombin time (IQR-s)	11.4 (10.7, 12.4)	11.3 (10.6, 12.2)	11.9 (11.1, 13.4)	< 0.0001	12.4 (11.3, 13.9)	11.6 (10.0, 12.6)	14 (12.4, 17.5)	< 0.0001
Distribution, *n* (%)								
<10.5	198 (19.1)	163 (20.4)	35 (14.7)	< 0.0001	38 (11.9)	34 (18.4)	4 (3.0)	< 0.0001
10.5–13.5	726 (70.0)	580 (72.6)	146 (61.3)		183 (57.4)	131 (70.8)	52 (38.8)	
>13.5	113 (10.9)	56 (7.0)	57 (24.0)		98 (30.7)	20 (10.8)	78 (58.2)	
Activated-partial thromboplastin time (IQR-s)	27.6 (24.3, 32.2)	27 (23.9, 31.1)	29.9 (25.7, 35.8)	< 0.0001	30 (25.0, 35.8)	29 (24.7, 34.3)	33.4 (26.1, 38.9)	0.0006
Distribution, *n* (%)								
<21	64 (6.2)	47 (5.9)	17 (7.3)	< 0.0001	21 (6.7)	14 (7.7)	7 (5.4)	< 0.0001
21–37	870 (84.2)	699 (87.5)	171 (73.1)		228 (73.1)	148 (80.9)	80 (62.0)	
>37	99 (9.6)	53 (6.6)	46 (19.6)		63 (20.1)	21 (11.4)	42 (32.6)	
Thrombin time (IQR-s)	17.8 (16.7, 20.6)	17.7 (16.7, 20.0)	18.4 (17.1, 22.4)	< 0.0001	18.4 (17.1, 21.7)	18.3 (17.1, 21.3)	18.4 (17.1, 23.0)	0.5313
Distribution, *n* (%)								
<13	8 (0.8)	8 (1.0)	0 (0.0)	0.0044	0 (0.0)	0 (0.0)	0 (0.0)	0.4132
13–21	782 (75.7)	618 (77.4)	164 (70.1)		224 (72.0)	135 (73.8)	89 (69.5)	
>21	243 (23.5)	173 (21.6)	70 (29.9)		87 (28.0)	48 (26.2)	39 (30.5)	
D-dimer (IQR-μg/mL)	0.8 (0.4, 1.9)	0.74 (0.4, 1.4)	1.38 (0.5, 9.4)	< 0.0001	2.21 (0.7, 18.1)	0.95 (0.5, 2.8)	17.83 (4.5, 56.5)	< 0.0001
Distribution, *n* (%)								
≤ 0.5	322 (31.9)	268 (34.5)	54 (23.2)	< 0.0001	55 (17.5)	51 (27.9)	4 (3.1)	< 0.0001
0.5–1	271 (26.8)	227 (29.2)	44 (18.9)		52 (16.6)	43 (23.5)	9 (6.9)	
>1	417 (41.3)	282 (36.3)	135 (57.9)		207 (65.9)	89 (48.6)	118 (90.0)	
Total bilirubin (IQR-μmol/L)	12.7 (9.9, 17.0)	12.1 (9.6, 15.6)	16 (11.7, 24.9)	< 0.0001	16.7 (11.9, 26.4)	14.05 (11.0, 18.4)	24.9 (16.6, 36.1)	< 0.0001
Distribution, *n* (%)								
≤ 26	954 (91.8)	777 (95.8)	177 (77.6)	< 0.0001	228 (74.5)	155 (90.1)	73 (54.5)	< 0.0001
>26	85 (8.2)	34 (4.2)	51 (22.4)		78 (25.5)	17 (9.9)	61 (45.5)	
Alanine aminotransferase ( (IQR-U/L)	41.5 (25.0, 64.0)	38 (23.0, 60.0)	51 (34.0, 83.0)	< 0.0001	50 (32.0, 79.0)	50 (33.0, 75.0)	47 (31.0, 84.0)	0.7016
Distribution, *n* (%)								
≤ 40	524 (49.2)	436 (52.9)	88 (36.5)	< 0.0001	123 (38.3)	72 (38.7)	51 (37.8)	0.8654
>40	542 (50.8)	389 (47.1)	153 (63.5)		198 (61.7)	114 (61.3)	84 (62.2)	
Aspartate aminotransferase (IQR-U/L)	34 (26.0, 49.0)	31 (24.0, 44.0)	46.5 (34.0, 72.0)	< 0.0001	48 (35.0, 74.0)	40 (31.0, 57.0)	58 (44.0, 109.0)	< 0.0001
Distribution, *n* (%)								
≤ 40	679 (63.7)	584 (70.9)	95 (39.3)	< 0.0001	118 (36.5)	96 (51.6)	22 (16.1)	< 0.0001
>40	387 (36.3)	240 (29.1)	147 (60.7)		205 (63.5)	90 (48.4)	115 (83.9)	
Albumin (IQR-g/L)	31.7 (28.5, 35.0)	32.4 (29.6, 35.7)	28.3 (26.0, 31.5)	< 0.0001	28 (25.5, 30.7)	29.5 (27.4, 32.3)	26.2 (24.3, 28.3)	< 0.0001
Distribution, *n* (%)								
<40	1024 (95.9)	780 (94.7)	244 (100.0)	0.0003	326 (100.0)	186 (100.0)	140 (100.0)	1
40–55	41 (3.8)	41 (5.0)	0 (0.0)		0 (0.0)	0 (0.0)	0 (0.0)	
>55	3 (0.3)	3 (0.3)	0 (0.0)		0 (0.0)	0 (0.0)	0 (0.0)	
Blood urea nitrogen (IQR-mmol/L)	5 (4.0, 6.4)	4.8 (3.8, 5.8)	6.5 (5.0, 10.2)	< 0.0001	7.2 (5.4, 11.7)	6.1 (4.7, 7.7)	13.2 (7.7, 20.3)	< 0.0001
Distribution, *n* (%)								
<3.1	81 (7.6)	74 (9.0)	7 (2.9)	< 0.0001	7 (2.2)	7 (3.8)	0 (0.0)	< 0.0001
3.1–8	838 (78.6)	688 (83.6)	150 (61.7)		177 (55.5)	139 (75.1)	38 (28.4)	
>8	147 (13.8)	61 (7.4)	86 (35.4)		135 (42.3)	39 (21.1)	96 (71.6)	
Serum creatinine (IQR-umol/L)	72.4 (59.4, 87.2)	70.9 (59.0, 83.0)	78.8 (62.5, 104.0)	< 0.0001	79.6 (63.0, 109.8)	73.9 (59.5, 91.6)	107.8 (69.2, 196.7)	< 0.0001
Distribution, *n* (%)								
>133	65 (6.1)	24 (2.9)	41 (16.9)	< 0.0001	60 (18.9)	8 (4.3)	52 (39.4)	< 0.0001
≤ 133	995 (93.9)	794 (97.1)	201 (83.1)		257 (81.1)	177 (95.7)	80 (60.6)	
Creatine kinase (IQR-U/L)	76 (50.0, 141.0)	71 (49.0, 123.0)	123 (54.0, 247.0)	< 0.0001	124.5 (55.5, 274.5)	89 (48.0, 196.0)	240 (101.0, 553.0)	< 0.0001
Distribution, *n* (%)								
<50	235 (24.4)	190 (25.2)	45 (21.5)	< 0.0001	53 (19.5)	46 (27.7)	7 (6.6)	< 0.0001
50–310	640 (66.5)	517 (68.6)	123 (58.9)		159 (58.4)	102 (61.5)	57 (53.8)	
>310	88 (9.1)	47 (6.2)	41 (19.6)		60 (22.1)	18 (10.8)	42 (39.6)	
Creatine kinase isoenzyme MB (IQR-U/L)	13 (10.0, 17.0)	13 (10.0, 16.0)	17 (13.0, 24.0)	< 0.0001	18 (14.0, 27.0)	15 (12.0, 20.0)	24 (18.0, 47.0)	< 0.0001
Distribution, *n* (%)								
≤ 24	921 (90.8)	747 (95.3)	174 (75.7)	< 0.0001	213 (70.5)	149 (84.7)	64 (50.8)	< 0.0001
>24	93 (9.2)	37 (4.7)	56 (24.3)		89 (29.5)	27 (15.3)	62 (49.2)	
Serum prealbumin (IQR-g/L)	132 (85.0, 191.0)	144 (98.0, 201.0)	86 (48.0, 132.0)	< 0.0001	78 (44.5, 122.5)	105.5 (70.5, 152.5)	48.5 (29.5, 75.0)	< 0.0001
Distribution, *n* (%)								
<200	799 (77.9)	588 (74.0)	211 (91.3)	< 0.0001	286 (92.9)	160 (88.9)	126 (98.4)	0.0013
200–430	227 (22.1)	207 (26.0)	20 (8.7)		22 (7.1)	20 (11.1)	2 (1.6)	
Serum amyloid protein A (IQR-mg/L)	186 (28.9, 272.3)	151.6 (20.6, 259.1)	242.4 (177.4, 284.0)	< 0.0001	246.45 (180.4, 284.0)	241.2 (132.4, 284.0)	260.1 (188.9, 284.0)	0.0103
Distribution, *n* (%)								
≤ 10	150 (16.9)	140 (20.0)	10 (5.4)	< 0.0001	11 (4.3)	9 (6.0)	2 (1.9)	0.2075
>10	737 (83.1)	560 (80.0)	177 (94.6)		245 (95.7)	142 (94.0)	103 (98.1)	
C-reactive-protein (IQR-mg/L)	25.6 (4.9, 79.1)	18.4 (3.8, 54.4)	86.25 (22.3, 160.0)	< 0.0001	102.5 (37.6, 160.0)	52.4 (12.1, 103.0)	160 (124.2, 177.1)	< 0.0001
Distribution, *n* (%)								
≤ 6.9	287 (30.2)	259 (35.4)	28 (12.8)	< 0.0001	31 (10.7)	28 (17.4)	3 (2.3)	< 0.0001
>6.9	663 (69.8)	473 (64.6)	190 (87.2)		258 (89.3)	133 (82.6)	125 (97.7)	
Serum ferritin (IQR-ng/mL)	377.72 (133.72, 723.96)	344.66 (136.53, 625.70)	557.58 (79.26, 1264.47)	0.0002	618.13 (150.31, 1503.90)	647.98 (245.35, 1193.72)	616.55 (38.68, 2000.00)	0.8666
Distribution, *n* (%)								
<21.8	35 (5.0)	27 (5.2)	8 (4.4)	0.0931	9 (3.6)	5 (3.6)	4 (3.6)	0.1069
21.8-274.6	247 (35.1)	192 (36.9)	55 (30.0)		71 (28.5)	32 (23.4)	39 (34.8)	
> 274.6	421 (59.9)	301 (57.9)	120 (65.6)		169 (67.9)	100 (73.0)	69 (61.6)	
Interleukin-6 (IQR-pg/mL)	14.0 (7.8, 398.8)	14.6 (7.8, 354.4)	13.3 (8.0, 648.4)	0.1783	13.9 (8.4, 660.9)	10.5 (7.2, 458.0)	31.9 (11.1, 1487.0)	< 0.0001
Distribution, *n* (%)								
≤ 7	28 (3.7)	20 (3.4)	8 (4.6)	0.4741	8 (3.4)	5 (4.0)	3 (2.8)	0.8896
> 7	729 (96.3)	563 (96.6)	166 (95.4)		226 (96.6)	121 (96.0)	105 (97.2)	
Radiologic findings								
Abnormalities, *n* (%)								
Ground-glass opacity	958 (92.3)	734 (91.9)	224 (93.7)	0.3444	293 (93.3)	176 (94.1)	117 (92.1)	0.4881
Pulmonary consolidation	171 (16.5)	106 (13.3)	65 (27.2)	< 0.0001	88 (28.0)	49 (26.2)	39 (30.7)	0.383
Pulmonary interstitial abnormalities	646 (62.3)	471 (59.0)	175 (73.2)	< 0.0001	229 (72.9)	138 (73.8)	91 (71.7)	0.6749
Pneumothorax	26 (2.5)	18 (2.3)	8 (3.4)	0.3437	13 (4.1)	6 (3.2)	7 (5.5)	0.3147
Pleural effusion	44 (4.2)	33 (4.1)	11 (4.6)	0.7505	16 (5.1)	10 (5.4)	6 (4.7)	0.8053

*SOFA* Sequential Organ Failure Assessment, *APACHEII* Acute Physiology and Chronic Health Evaluation II

### Radiologic and laboratory findings

A total of 1027 (92.3%) patients had findings of ground-glass opacity on radiographic imaging, 700 (63.0%) patients had interstitial abnormalities. Complex radiologic features such as consolidation (27.2% vs. 13.3%, *p *< 0.0001) and interstitial changes (73.2% vs. 59.0%, *p *< 0.0001) and abnormal laboratory results such as hyperleukocytosis (38.9% vs. 10.9%, *p *< 0.0001), lymphocytopenia (49.6% vs. 18.8%, *p *< 0.0001), thrombocytopenia (23.5% vs. 4.1%, *p *< 0.0001) and hypercoagulability (APTT, PT, TT, D-dimer, all *p *< 0.0001) occurred more in progressors than in non-progressors. There were no significant differences in IL-6 level between the two groups (14.6 pg/ml in non-progressors vs. 13.3 pg/ml in progressors, *p *= 0.178). Abnormal results of laboratory tests (e.g., hyperleukocytosis [71.7% vs. 27.4%, *p *< 0.0001], lymphocytopenia [72.9% vs. 38.3%, *p *< 0.0001], lower CD4 count [211/μL vs. 353/μL, *p *= 0.0003], thrombocytopenia [52.9% vs. 8.1%, *p *< 0.0001], hypercoagulability especially elevated D-dimer [90.1% vs. 48.6%, *p *< 0.0001]) were also common in non-survivors (Tables 1, 2).

### Treatment

During hospitalization, most (*n* = 987, 82.9%) of patients received oxygen therapy, including nasal cannula (*n* = 792, 66.6%), mask oxygen inhalation (*n* = 27, 2.3%), high-flow nasal cannula (*n* = 60, 5.0%), non-invasive mechanical ventilation (*n* = 62, 5.2%), invasive mechanical ventilation (*n* = 42, 3.5%) and ECMO (*n* = 4, 0.3%, Table 2). 10.2% (*n* = 16) severe patients who suddenly died treated with nasal cannula, 4.5% (*n* = 7) dead severe patients treated with mask oxygen inhalation, 22.9% (*n* = 36) dead severe patients treated with high-flow nasal cannula, 36.9% (*n* = 58) dead severe patients treated with non-invasive mechanical ventilation, 22.9% (*n* = 36) with invasive mechanical ventilation and 2.6% (*n* = 4) with ECMO. Among not severe patients, 259 (99.2%) patients received oxygen therapy in the progression group vs. 640 (76.1%) in the non-progression group (*p *< 0.0001). Compared with the survivors with severe disease, significantly more non-survivors received non-invasive mechanical ventilation, invasive mechanical ventilation and ECMO (62.4% vs. 4.7%, *p *< 0.0001) and antiviral treatment (71.8% vs. 43.3% *p *< 0.0001). More remarkably, there were more non-survivors treated with glucocorticoids (59.8% vs. 39.7%, *p *= 0.0005) among severe patients (Table 4).

**Table 4:** Treatments during hospital stay and clinical outcomes of the study patients

	Not severe patients at admission (*n* = 1102)	Non-progressors (*n* = 841)	Progressors (*n* = 261)	*p* value	Severe patients (*n* = 349)	Survivor (*n* = 192)	Non-survivor (*n* = 157)	*p* value
Treatments, *n* (%)								
Antibiotic	905 (87.1)	690 (84.9)	215 (95.1)	< 0.0001	287 (95.4)	169 (97.1)	118 (92.9)	0.0865
Antifungal	44 (4.2)	22 (2.7)	22 (9.7)	< 0.0001	28 (9.3)	13 (7.5)	15 (11.8)	0.2005
Antiviral	654 (63.0)	501 (61.6)	153 (67.7)	0.0943	180 (59.8)	125 (71.8)	55 (43.3)	< 0.0001
Glucocorticoids	251 (24.2)	144 (17.7)	107 (47.4)	< 0.0001	145 (48.2)	69 (39.7)	76 (59.8)	0.0005
Oxygen therapy, *n* (%)				< 0.0001				< 0.0001
None	203 (18.4)	201 (23.9)	2 (0.8)		2 (0.6)	2 (1.0)	0	
Nasal cannula	792 (71.9)	634 (75.4)	158 (60.5)		158 (45.3)	142 (74.0)	16 (10.2)	
Mask oxygen	17 (1.5)	4 (0.5)	13 (5.0)		23 (6.6)	16 (8.3)	7 (4.5)	
High-flow nasal cannula	25 (2.3)	1 (0.1)	24 (9.2)		59 (16.9)	23 (12.0)	36 (22.9)	
Non-invasive mechanical ventilation	34 (3.1)	0 (0.0)	34 (13.0)		62 (17.8)	4 (2.1)	58 (36.9)	
Invasive mechanical ventilation	28 (2.5)	1 (0.1)	27 (10.3)		41 (11.8)	5 (2.6)	36 (22.9)	
ECMO	3 (0.3)	0 (0.0)	3 (1.2)		4 (1.2)	0 (0.0)	4 (2.6)	
Outcomes								
Duration of MV (IQR), days	4 (2.0, 8.0)	0	4 (2.0, 8.0)		5 (2.0, 8.0)	6 (5.0, 9.0)	4 (2.0, 8.0)	0.1563
Duration of ICU stay (IQR), days	0	0	6 (3.0, 10.0)		6 (3.0, 10.5)	7 (4.0, 11.0)	5 (2.0, 9.0)	0.0522
Duration of in-hospital stay (IQR), days	11 (8.00, 15.00)	11 (8.0, 14.0)	12 (8.0, 16.0)	0.0021	11 (7, 16)	14 (10.0, 18.0)	8 (4.0, 12.0)	< 0.0001
In-hospital mortality, *n* (%)	91 (8.26)	0 (0.0)	91 (34.9)	< 0.0001	157 (45.0)	0 (0.0)	157 (100.0)	< 0.0001

*ECMO* extracorporeal membrane oxygenation, *ICU* intensive care unit, *MV* mechanical ventilation

Nine hundred and seventy-seven (87.7%) patients were treated with empirical antibiotic treatment (*e.g.,* ceftriaxone, moxifloxacin and azithromycin), 681 (61.1%) antiviral therapy (*e.g.,* oseltamivir, ganciclovir, lopinavir/ritonavir, arbidol and interferon), and 289 (25.9%) glucocorticoids. Empirical antibiotic treatment, antiviral therapy and glucocorticoids on admission were also given more commonly to progressors than to non-progressors (Tables 2, 4).

### Outcomes

**Fig. 2:**
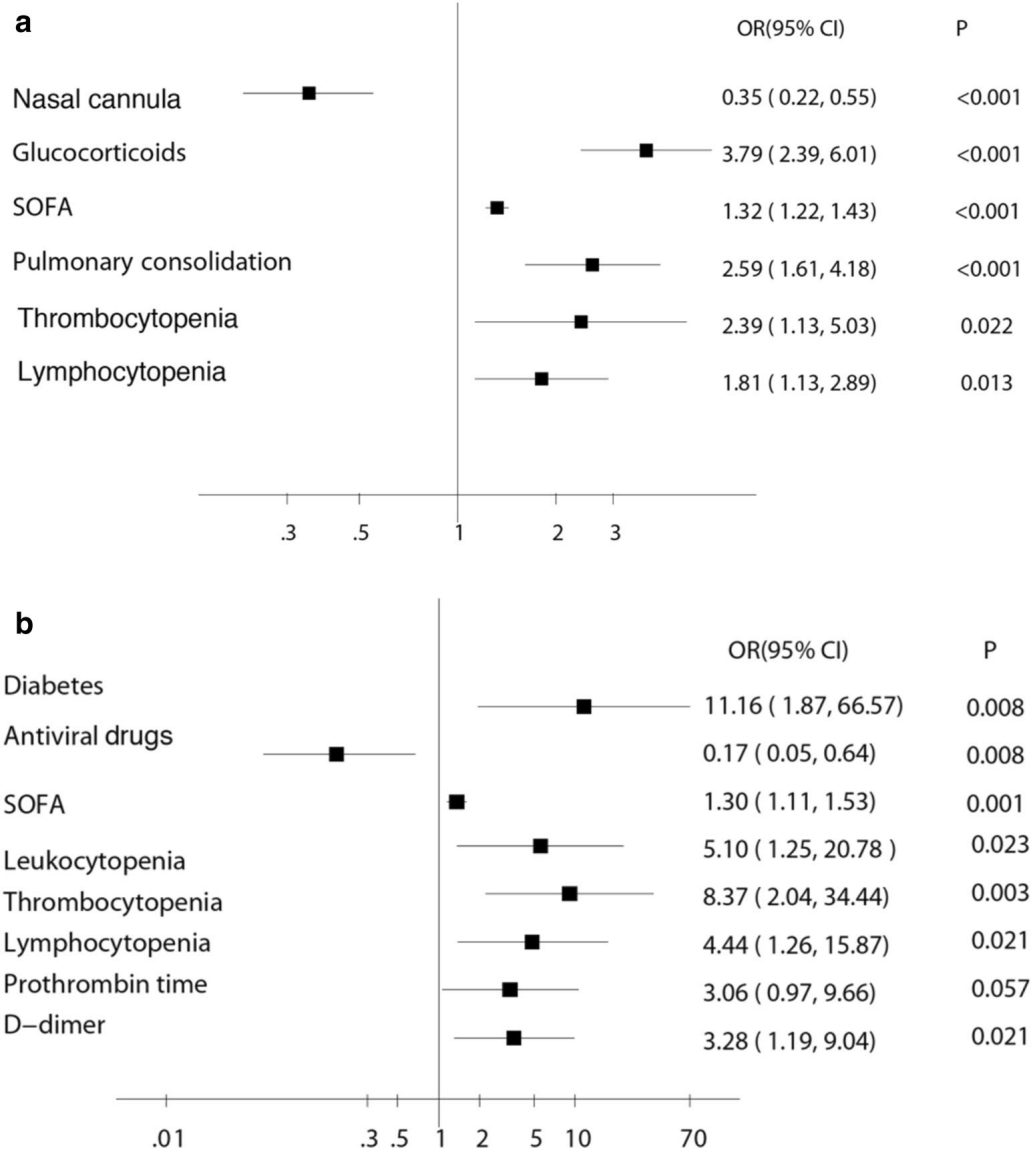
Odds ratios for risk factors associated with in-hospital progression **a** in COVID-19 patients and in-hospital mortality **b** in severe COVID-19 patients. SOFA: Sequential Organ Failure Assessment. IMV: invasive mechanical ventilation. Nasal cannula, glucocorticoids treatment, IMV and antiviral drugs during hospital stay; SOFA score, pulmonary consolidation, leukocytosis, thrombocytopenia and lymphocytopenia, prothrombin time and D-dimer on admission

Two hundred and sixty-one (22.7%) patients without severe condition on admission progressed to severe pneumonia. To analyze the associations between patients’ variables and disease development, a multivariate analysis was performed. As shown in Fig. 2a, independent risk factors for development from not severe to severe disease were presence of pulmonary consolidation (OR 2.59, 95% CI 1.61–4.18, *p *< 0.001), SOFA score on admission (OR 1.32, 95% CI 1.22–1.43, *p *< 0.001), lymphocytopenia (OR 1.81, 95% CI 1.13–2.89, *p *= 0.013) and thrombocytopenia (OR 2.39, 95% CI 1.13–5.03, *p *= 0.022). Of note, the deterioration of disease cannot be prevented by glucocorticoids (OR 3.79, 95% CI 2.39–6.01, *p *< 0.001), but could be prevented by NIV. Independent risk factors for death among all the included patients are shown in Additional file 3: Table S2.

**Fig. 3:**
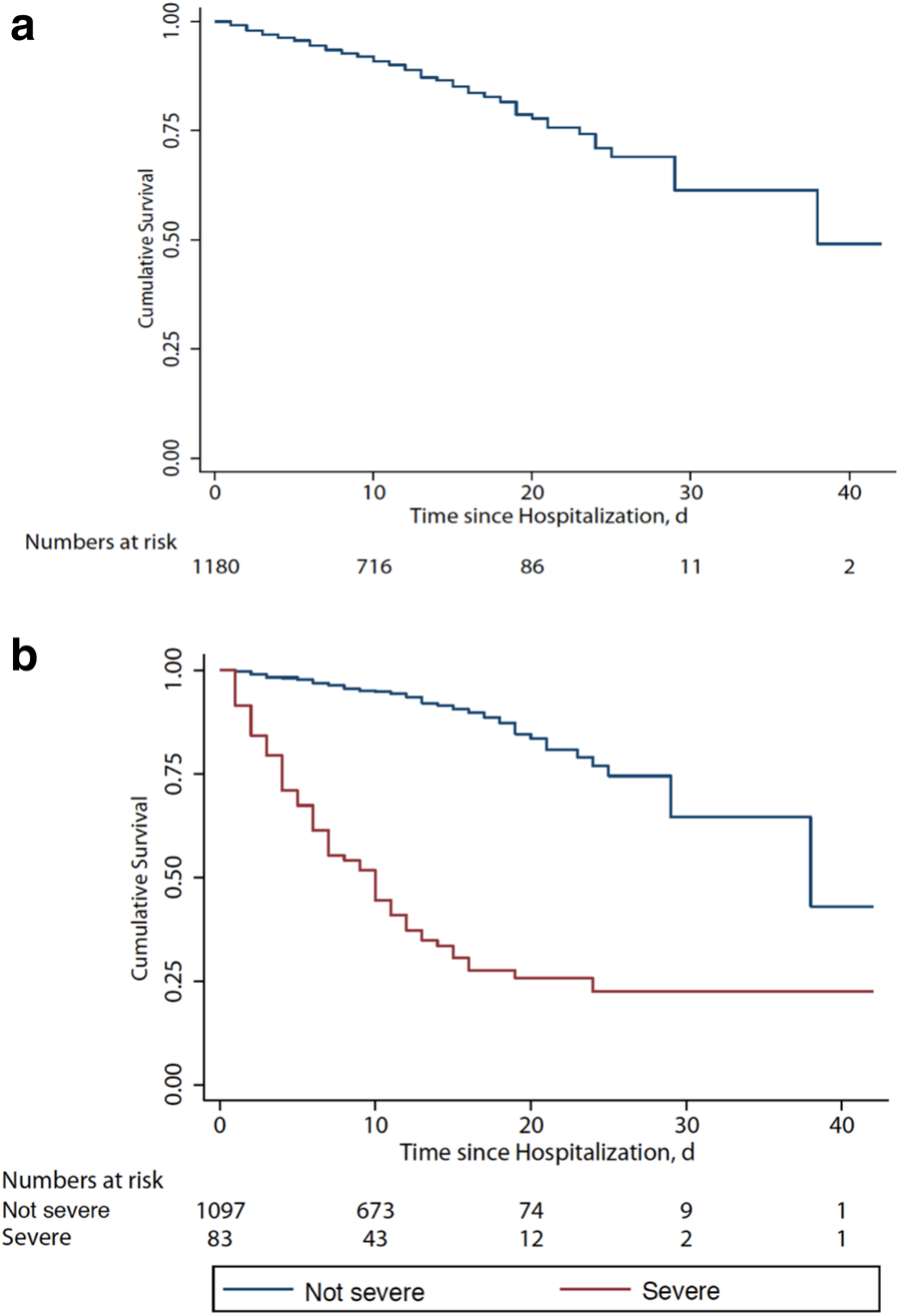
Survival curve in with coronavirus disease. **a** In all enrolled patients. **b** In not severe and severe patients. Nine patients died on admission as a result of unsuccessful rescue efforts

**Fig. 4:**
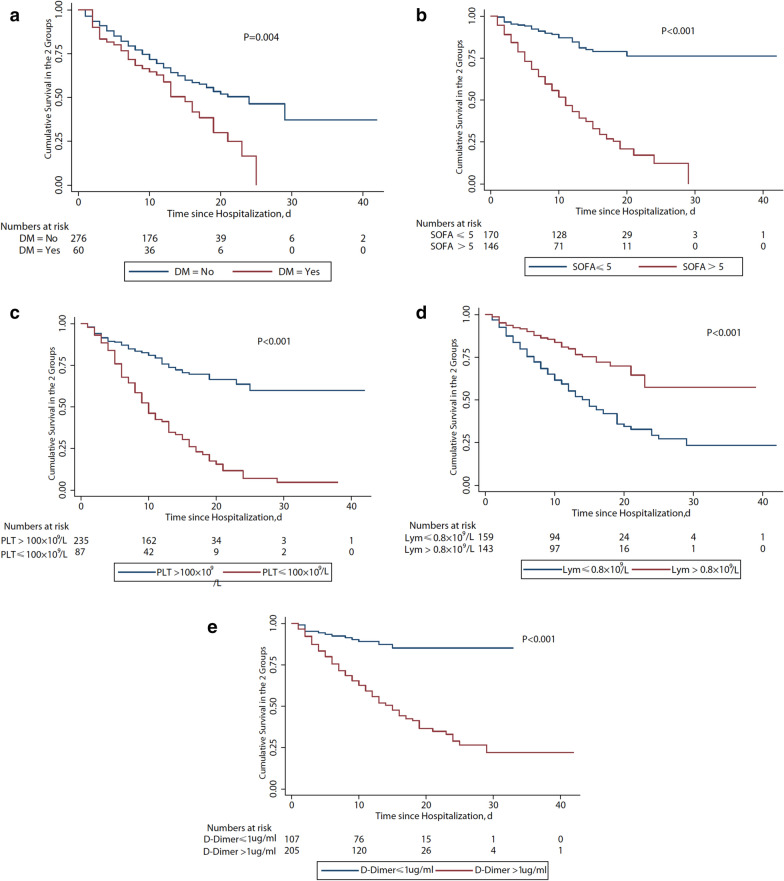
Cumulative survival curves among severe COVID-19 patients. **a** With DM and without DM; **b** SOFA score > 5 and SOFA score ≤ 5 on admission; **c** PLT counts on admission; **d** Lym counts on admission; **e** D-dimmer on admission. *DM* diabetes mellitus, *SOFA* Sequential Organ Failure Assessment, *PLT* platelet; *Lym* lymphocyte

**Fig. 5:**
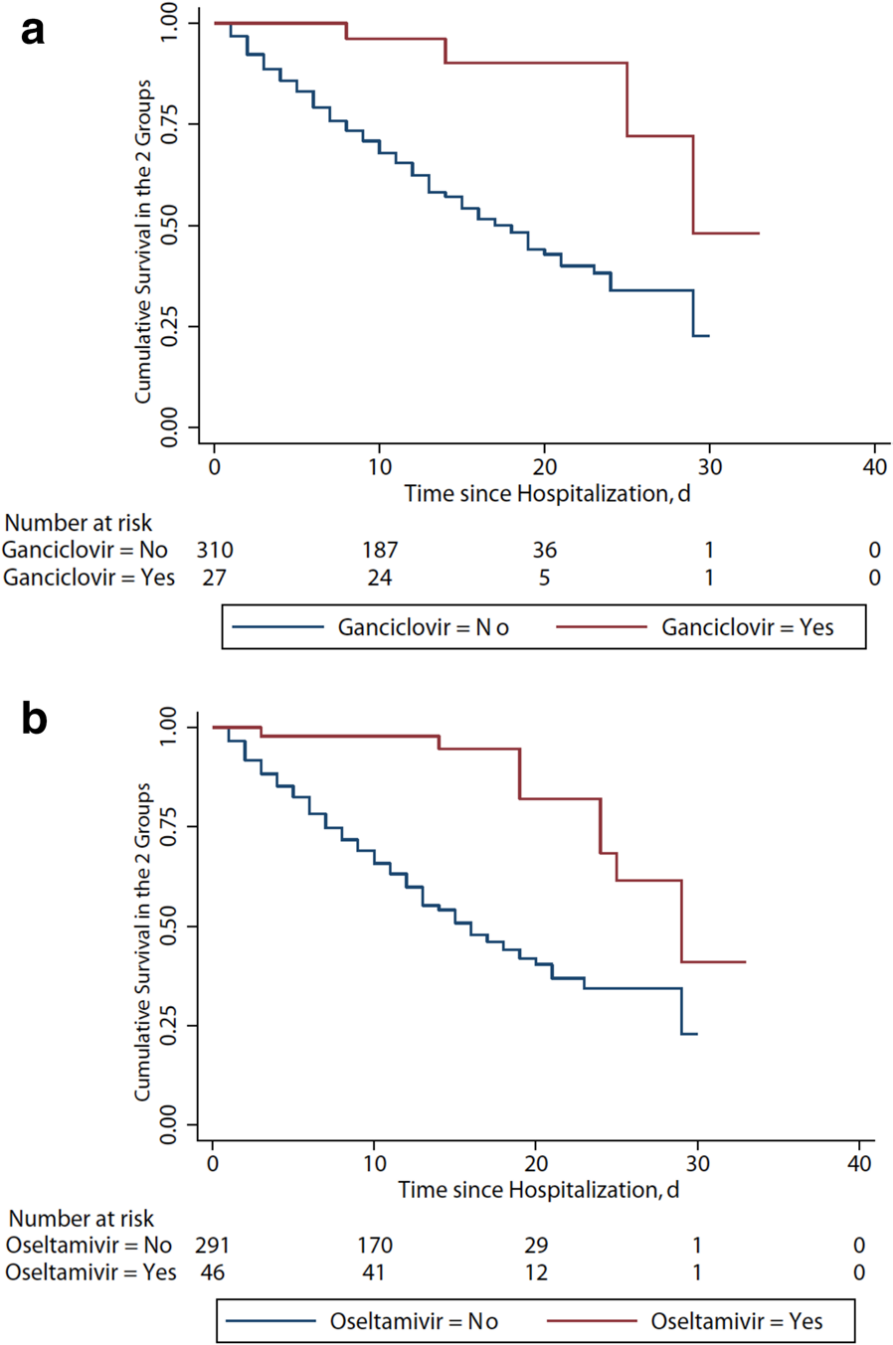
Cumulative survival curve in severe COVID-19 patients with oseltamivir or ganciclovir. **a** Use of ganciclovir during hospital stay reduced the risk of death (hazard ratio, 0.20; 95% CI 0.07–0.55; *p *< 0.001). **b** Use of oseltamivir during hospital stay reduced the risk of death (hazard ratio, 0.21; 95% CI 0.10–0.43; *p *< 0.001)

Among severe patients, 192 survived and 157 died. Figure 3b shows the survival curve. The risk of death was more than 11 times higher in patients with diabetes than those without diabetes (OR 11.16, 95% CI 1.87–66.57, *p *= 0.008; Fig. 2b). Other significant independent risk factors for mortality were on admission SOFA score (OR 1.30, 95% CI 1.11–1.53, *p *= 0.001), leukocytopenia (OR 5.10, 95% CI 1.25–20.78, *p *= 0.023), lymphocytopenia (OR 4.44, 95% CI 1.26–15.87, *p *= 0.021), thrombocytopenia (OR 8.37, 95% CI 2.04–34.44, *p *= 0.003) and elevated D-dimer (OR 3.28, 95% CI 1.19–9.04, *p *= 0.021, Fig. 4). Survival curves of severe patients according to those mortality predictors are shown in Fig. 5. In a multivariate analysis, antiviral treatment during hospital stay was negatively associated with death (OR 0.17, 95% CI 0.05–0.64, *p *= 0.008) among severe patients with COVID-19. In order to figure out which of them made the major contribution to prolong survival, we conducted survival analysis and found the administration of oseltamivir (HR 0.21, 95% CI 0.10–0.43; *p *< 0.001) or ganciclovir (HR 0.20, 95% CI 0.07–0.55, *p *< 0.001) appeared to have reduced the risk of death in severe patients (Fig. 5).

**Fig. 6:**
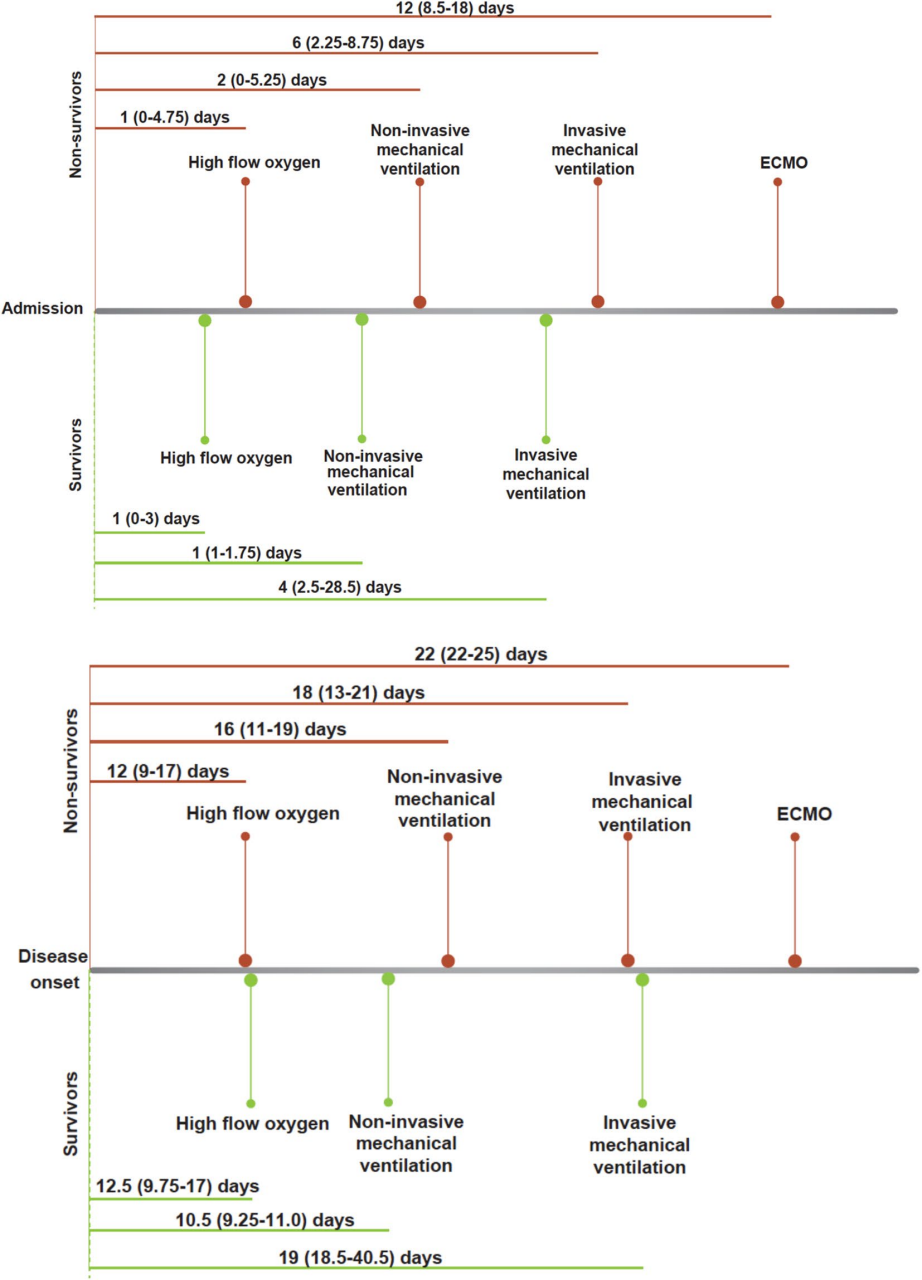
Time interval between admission and disease onset to respiratory supports. Respiratory supports include high-flow oxygen, non-invasive mechanical ventilation, invasive mechanical ventilation and ECMO. *ECMO* extracorporeal membrane oxygenation

The time interval from disease onset to high-flow nasal cannula, non-invasive mechanical ventilation, invasive mechanical ventilation in survivors with severe disease was 12 day (IQR, 10–17), 11 days (IQR, 9–11), 19 days (IQR 19–41), respectively. However, the time interval from admission to high-flow nasal cannula was 12 days (IQR, 9–17), to non-invasive mechanical ventilation was 16 days (IQR, 11–19), to invasive mechanical ventilation was 18 days (IQR, 13–21) and to ECMO was 22 days (IQR, 22–25) in non-survivors with severe disease. The time interval from admission to high-flow nasal cannula was 1 day (IQR, 0–3), to non-invasive mechanical ventilation was 1 day (IQR, 1–2), to invasive mechanical ventilation was 4 days (IQR, 3–29) in the survivors with severe disease. In the non-survivors with severe disease, the time interval from disease onset to high-flow nasal cannula, non-invasive mechanical ventilation, invasive mechanical ventilation and ECMO was 1 day (IQR, 0–5), 2 days (IQR, 0–5), 6 days (IQR 2–9) and 12 (9–18), respectively (Fig. 6).

## Discussion

This retrospective cohort study included a very large number of COVID-19 patients reported clinical outcomes and potential risk factors for development from not severe to severe manifestations after admission, as well as those who progressed from severe disease to death. In particular, higher SOFA score, lymphocytopenia on admission were independent risk factors for development to severe manifestations and death. On admission, level of D-dimer greater than 1 μg/L and diabetes were associated with higher risks of in-hospital death in patients with severe COVID-19. Administration of glucocorticoids seemed to increase the risk of deterioration to severe disease after admission. Anti-virus drugs (ganciclovir, oseltamivir) seemed to be associated with less deterioration from not severe to severe disease and from severe disease to death. Moreover, early IMV may be helpful to decrease mortality in severe patients. The risk factors presented in the current study may be helpful for clinicians to early identify patients who will probably progress to severe illness during in-hospital stay. Early interventions could be given to decrease mortality in COVID-19 patients with abnormal biological results. However, the benefits of anti-virus drugs should be interpreted with caution in the absence of data from randomized controlled studies.

COVID-19 patients with Acute Respiratory Distress Syndrome (ARDS) are severe, therefore the respiratory support of COVID-19 patients is essential to decrease mortality. However, there is still controversy regarding the prognosis of COVID-19 after the initiation of mechanical ventilation [12]. Also it is still necessary to explore that if invasive mechanical ventilation could improve outcome of COVID-19 patients when compared to non-invasive mechanical ventilation [13]. The present results show that time interval from admission to non-invasive mechanical ventilation in survivors with severe disease was shorter compared with that in non-survivors with severe disease. COVID-19 patients may acquire prognostic benefit from early respiratory support. Since frequent monitoring is needed during process of non-invasive mechanical ventilation, non-invasive mechanical ventilation treatment should be used with caution in resource-limited settings.

The SOFA score is an important marker to indicate the severity of multiple organ dysfunction [14]. Although the common pathogen to cause sepsis or septic shock is bacteria, virus also causes sepsis particularly in community-acquired pneumonia [15]. In the present study, higher SOFA score on admission increases the risk of death of severe COVID-19 patients. This is consistent with previous results [16]. A recent study suggested that the spike protein of SARS-CoV-2 has a strong affinity to human angiotensin-converting enzyme 2 (ACE 2) for host infection [17]. The SARS-CoV-2 spike protein directly binds with the host cell surface ACE2 receptor facilitating virus entry and replication. ACE2 was expressed in many organs, and is rich in lungs, heart, kidneys and intestine [18]. Therefore, organ injuries caused by SARS-CoV-2 are extensive and become highly lethal because the virus deregulates an organ protective pathway [19].

Presence of comorbidities was found to be an independent predictor of poor outcome in our patients. Previous history of cardiovascular diseases (CVD) is independent associated with increased all-cause mortality and in-hospital deterioration COVID-19 patients [20]. This may be related with enhanced severity of an underlying CVD by occurrence of COVID-19. The prognostic effect of diabetes mellitus has been previously reported in other cohorts of patients with Middle East respiratory syndrome (MERS) [21] and SARS [22]. The prognostic relationship between diabetes mellitus and acute viral respiratory infections has been already identified [23]. Diabetes mellitus has also been identified as a prognostic factor for death in patients with community-acquired pneumonia (CAP) [24]. This is consistent with the fact that diabetes could predispose patients to be immunologically vulnerable [25]. The innate immunity is impaired through suppression of the number and function of T cells and neutrophils in diabetic patients [26]. Secondary infections are common in diabetic patients due to impaired inflammatory and immune biomarker profiles [27]. The counts of T cells including CD3 T cells, CD4 T cells and CD8 T cells decreased in non-survivors of COVID-19 in the present study. All these findings indirectly argue in favor of the role of diabetes mellitus as a prognostic factor in our patients. However, the direct influence of diabetes mellitus on SARS-Cov-2 infection still needs to be elucidated.

Lymphocytopenia was found as a potential predictor for disease development and death. Thrombocytopenia and leukocytosis also occurred in the severe cases. This may suggest that enhanced inflammation and cytokine storm started from the initial stage. These biological abnormalities were previously observed in patients with severe MERS-CoV-infected patients [28]. Cytokines are mostly secreted from neutrophils. In patients with MERS, lung injury was correlated with migration of neutrophils and macrophages from peripheral blood to extensive pulmonary [29, 30]. ARDS caused by cytokine storm was a leading cause of death in patients with Middle East respiratory syndrome [31]. In our study, only serum IL-6 level has been quantified in some of the COVID-19 patients. However, it is difficult to clarify the influence of cytokine storm on outcome due to missing of IL-6 and other cytokines data.

D-dimer produced by fibrin degradation, reflects the severity of hyper-coagulable state [32]. Coagulation could be activated to enhance physiological response to several infections [33]. Microvascular failure and subsequent multiple organ failure could be alleviated through inhibiting activation of coagulation and subsequently improve outcome during systemic hyperinflammation and fulminant sepsis [34]. D-dimer was previously found to be associated with pneumonia progression [35] and in-hospital mortality [36]. The association between elevated D-dimer level with lethal outcome of COVID-19 patients was also reported in a previous study [16]. ACE 2 is also expressed on vascular endothelial cells [37]. Thus, one can postulate that coagulation is activated due to high affinity of SARS-CoV-2 with vascular endothelial cells. This can potentially contribute to elevated D-dimer level.

This study has several limitations. First, some laboratory data were missing or not available due to the retrospective data extraction. It should be noted that if important laboratory parameters (such as cardiac troponin, lactic dehydrogenase) were not included in the multivariable analyses, it may cause bias for results. However, we used CK-MB as an alternative indicator of cardiac injury. In addition, we also performed a sensitivity analysis using multiple imputations to account for missing data. The results did not change significantly before or after multiple imputations. Second, benefits of anti-virus drugs on mortality were observed in this study, but we could not further analyze the reason. The mixed virus infection of COVID-19 patients administered with anti-virus drugs should be further explored. Third, although the current study included over 1100 patients from Wuhan Infectious Disease Hospital, still there is a lack of dynamic change for related indicators. Fourth, treatment with methylprednisolone was harmful for not severe patients, however, the dose and duration of methylprednisolone varied, detailed results failed to demonstrate. However, this was the largest cohort study of COVID-19 patients from Wuhan Infectious Disease Hospital until now. A large multi-center cohort study of patients with COVID-19 pneumonia needs to further explore the clinical characteristics and risk factors of the disease.

## Conclusions

In this cohort study, higher SOFA score and lymphocytopenia on admission could predict that not severe patients would develop severe disease in-hospital. Elevated D-dimer on admission, leukocytopenia, thrombocytopenia and diabetes were independent risk factors of in-hospital death in severe patients with COVID-19. These specific characteristics will help clinicians to clarify the progression and the poor prognosis of COVID-19 patients.

## Supplementary information

**Additional file 1.** The classification of COVID-19.

**Additional file 2: Table S1.** Major complications in survivors and non-survivors.

**Additional file 3: Table S2.** Multivariate analysis of risk factors associated with in-hospital death in COVID-19 patients.

## Data Availability

Not applicable for no datasets were generated or analyzed in our study.

## Abbreviations

COVID-19: Coronavirus disease 2019
SARS-Cov-2: Severe acute respiratory syndrome coronavirus 2
SOFA: Sequential Organ Failure Assessment
CDC: Center for Disease Control and Prevention
ARDS: Acute respiratory distress syndrome
WHO: World Health Organization
ECMO: Extracorporeal membrane oxygenation
CT: Computed tomographic
RT-PCR: Real-time reverse-transcriptase polymerase chain reaction
NIV: Non-invasive mechanical ventilation
IMV: Invasive mechanical ventilation
MERS: Middle East respiratory syndrome
CAP: Community-acquired pneumonia
