# Supplementary material for: Clinical outcomes of COVID-19 in Wuhan, China: a large cohort study
Source: Ann Intensive Care. 2020 Jul 31;10:99. doi: 10.1186/s13613-020-00706-3 (PMC7393341; doi:10.1186/s13613-020-00706-3)
Supplement: Supplementary file 1 — Additional file 1. The classification of COVID-19. [file 13613_2020_706_MOESM1_ESM.docx]

Severity of COVID-19:

1. Mild: Patients with mild clinical symptoms but without abnormal radiological findings.

2. Moderate: Patients present with fever, cough and other symptoms are with virus pneumonia imaging.

tomography.

3. Severe

Patients met one of below conditions:

(1) Respiratory distress, respiratory rate ≥ 30 per min;

(2) Oxygen saturation at room air at rest ≤ 93%;

(3) Oxygen index less than 300 mmHg.

4. Critically ill

Patients met one of below conditions:

(1) Respiratory failure with mechanical ventilation;

(2) Accompanied with shock ;

(3) Patients with organ dysfunction admitted into intensive care unit.
