# Supplementary material for: Clinical outcomes of COVID-19 in Wuhan, China: a large cohort study
Source: Ann Intensive Care. 2020 Jul 31;10:99. doi: 10.1186/s13613-020-00706-3 (PMC7393341; doi:10.1186/s13613-020-00706-3)
Supplement: Supplementary file 2 — Additional file 2: Table S1. Major complications in survivors and non-survivors. [file 13613_2020_706_MOESM2_ESM.docx]

**Additional file 1: Table S1. Major complications in survivors and non-survivors**

| Complications | All patients  (n=1190) | Survivor  (n=1033) | Non-survivor  (n=157) | *p* value |
| --- | --- | --- | --- | --- |
| ARDS | 349(29.3) | 192(18.6) | 157(100.0) | <0.001 |
| Acute cardiac injury | 78(6.6) | 11(1.1) | 67(42.7) | <0.001 |
| Acute liver injury | 45(3.8) | 11(1.1) | 34(21.7) | <0.001 |
| Acute kidney injury | 46(3.9) | 7(0.7) | 39(24.8) | <0.001 |
| Shock | 75(6.3) | 4(0.4) | 71(45.2) | <0.001 |
| Data were presented as n(percentage).  ARDS=acute respiratory distress syndrome | | | | |
