# Supplementary material for: Clinical outcomes of COVID-19 in Wuhan, China: a large cohort study
Source: Ann Intensive Care. 2020 Jul 31;10:99. doi: 10.1186/s13613-020-00706-3 (PMC7393341; doi:10.1186/s13613-020-00706-3)
Supplement: Supplementary file 3 — Additional file 3: Table S2. Multivariate analysis of risk factors associated with in-hospital death in COVID-19 patients. [file 13613_2020_706_MOESM3_ESM.docx]

| Multivariable | p value | OR | 95% CI |
| --- | --- | --- | --- |
| Disease classification | <0.001 | 26.72 | 8.92,80.08 |
| Diabetes | 0.026 | 6.49 | 1.25,33.66 |
| Antiviral | 0.002 | 0.11 | 0.03,0.45 |
| SOFA | <0.001 | 1.43 | 1.18,1.73 |
| Thrombocytopenia | 0.001 | 10.42 | 2.54,42.74 |
| Lymphocytopenia | 0.041 | 0.24 | 0.06,0.94 |
| Hyperbilirubinemia | 0.042 | 4.31 | 1.05,17.67 |

Additional file 2: **table S2** Multivariate Analysis of risk factors associated with in-hospital death in COVID-19 patients
